# Hepatitis B: Model Systems and Therapeutic Approaches

**DOI:** 10.1155/2024/4722047

**Published:** 2024-05-07

**Authors:** Xiaoxiao Yu, Yating Gao, Xin Zhang, Longshan Ji, Miao Fang, Man Li, Yueqiu Gao

**Affiliations:** ^1^Laboratory of Cellular Immunity, Shuguang Hospital, Affiliated to Shanghai University of Traditional Chinese Medicine, Shanghai, China; ^2^Department of Hepatopathy, Shuguang Hospital, Affiliated to Shanghai University of Traditional Chinese Medicine, Shanghai, China; ^3^Institute of Infectious Diseases of Integrated Traditional Chinese and Western Medicine, Shanghai, China

## Abstract

Hepatitis B virus (HBV) infection is a major global health issue and ranks among the top causes of liver cirrhosis and hepatocellular carcinoma. Although current antiviral medications, including nucleot(s)ide analogs and interferons, could inhibit the replication of HBV and alleviate the disease, HBV cannot be fully eradicated. The development of cellular and animal models for HBV infection plays an important role in exploring effective anti-HBV medicine. During the past decades, advancements in several cell culture systems, such as HepG2.2.15, HepAD38, HepaRG, hepatocyte-like cells, and primary human hepatocytes, have propelled the research in inhibiting HBV replication and expression and thus enriched our comprehension of the viral life cycle and enhancing antiviral drug evaluation efficacy. Mouse models, in particular, have emerged as the most extensively studied HBV animal models. Additionally, the present landscape of HBV therapeutics research now encompasses a comprehensive assessment of the virus's life cycle, targeting numerous facets and employing a variety of immunomodulatory approaches, including entry inhibitors, strategies aimed at cccDNA, RNA interference technologies, toll-like receptor agonists, and, notably, traditional Chinese medicine (TCM). This review describes the attributes and limitations of existing HBV model systems and surveys novel advancements in HBV treatment modalities, which will offer deeper insights toward discovering potentially efficacious pharmaceutical interventions.

## 1. Introduction

Hepatitis B virus (HBV) has been prevalent worldwide for decades and is the primary cause of liver cirrhosis and liver cancer. The use of HBV vaccine has greatly decreased the incidence of HBV infection [1]. However, it is estimated that 292 million individuals continue with chronic carriage of HBV surface antigen [2], which is responsible for approximately half of the hepatocellular carcinoma incidences in the Asia–Pacific region [3]. HBV infection remains an international public health problem, which results in a high disease burden in most developing nations [4, 5]. Currently, nucleot(s)ide analogs (NAs) are one of the main antiviral therapeutic agents, primarily targeting viral replication. Another class of clinical first-line drugs is interferon, which mainly exerts antiviral effects through direct and indirect routes. However, the above antiviral therapies exhibit minimal influence on surface antigens and viral reservoirs. Most patients cannot achieve a functional cure for HBV infection with current therapy. Therefore, more research is urgently needed to better understand the pathogenesis of the disease and to improve its clinical management and outcome. Here, we summarize the progress of cell and animal models for supporting researches on HBV infection and explore the related mechanisms and the new therapeutic targets, which will provide more effective strategies for the resolution of chronic hepatitis B.

## 2. General Characteristics of HBV

**Figure 1:**
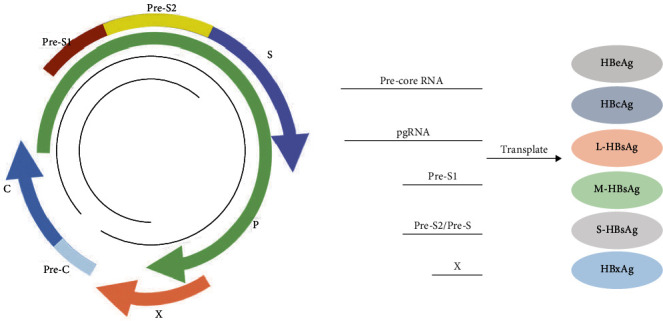
Structure of the HBV genome. A double-stranded circular DNA genome linked to a polymerase enzyme, surrounded by a nucleocapsid and three envelope proteins. It contains four open reading frames, i.e., the S, C, P, and X frames. The S frame, which contains the pre-S1, pre-S2, and S frames, can be transcribed into HBsAg. The C frame, which contains the pre-C and C frames, can be transcribed into HBcAg and HBeAg. The X frame can be transcribed into HBxAg. The P frame can be transcribed into DNA polymerase.

HBV was initially identified by Dane et al. [6], exhibiting a marked predilection for human hepatocytes. It contains a 3.2-kb relaxed circular DNA (rcDNA) strand [7–9]. As depicted in Figure 1, the Dane particle, the virus infectious agent, consists of a nucleocapsid, three envelope proteins, and a partly double-stranded circular DNA. The genome of HBV consists of four open reading frames: S, C, X, and P frames. The S open reading frame produces three proteins—large, middle, and small—collectively forming the HBsAg, a hallmark of HBV infection. The large transcript contains the pre-S1 domain, which serves as a template for L-HBsAg. The shorter transcripts contain the pre-S2 and S domains, which can be translated into M- and S-HBsAg, respectively. The C open reading frame is the source of both HBeAg and HBcAg, which indicates the active replication of HBV. HBeAg and HBcAg are translated from the pre-C and C transcripts, respectively, and the latter is also known as the pgRNA transcript. The X protein (HBx), which contributes to the persistence of cccDNA transcription and the progression of hepatocellular carcinoma, is encoded by the X open reading frame. Finally, the P frame encodes the essential enzymes for viral DNA synthesis, including DNA polymerase.

## 3. HBV Life Cycle

**Figure 2:**
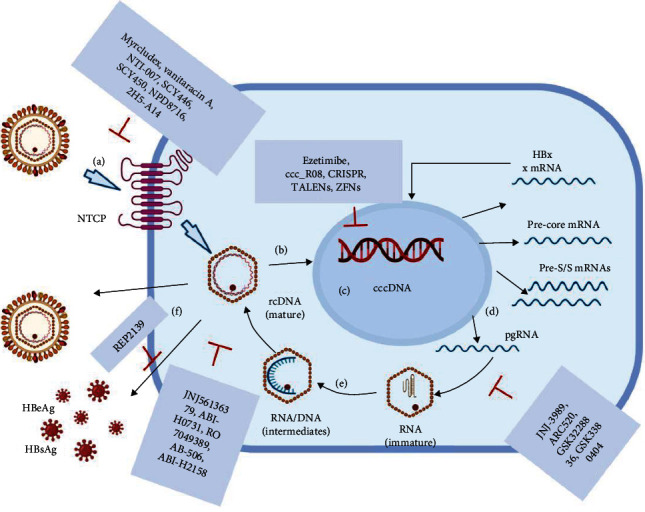
The life cycle of the hepatitis B virus and antiviral compounds. (a) The complete hepatitis B virus first binds to the sodium taurocholate cotransporting polypeptide (NTCP) receptor on the host cell via the pre-S1 domain of the hepatitis B surface antigen. Myrcludex, bacitracin, NTI-007, SCY446, SCY450, N, and PD8716 could inhibit the entry of the virus. (b) rcDNA is delivered into the nucleus. (c) The rcDNA is converted into a molecular DNA template (covalently closed circular DNA, cccDNA), and cccDNA is integrated into host cells. Ezetimibe, TALENs, ZFNs, and CRISPR/Cas9 nucleases inhibit the formation of cccDNA. (d) DNA is transcribed into viral RNAs, such as pgRNA, precure mRNA, pre-S/S mRNAs, and HBx mRNA. (e) pgRNA is reverse-transcribed into viral DNA. JNJ-3989 and ARC520 promote RNA degradation; GSK3228836 and GSK3380404 result in ribonuclease cleavage. (f) Mature nucleocapsids are bounded by large HBsAg molecules. The virus then acquires an envelope via the endosomal sorting complex, which is required for protein transportation. JNJ56136379, ABI-H0731, RO7049389, AB-506, and ABI-H2158 block the assembly nucleocapsid. REP2139 inhibited the release of HBsAg. Source: created with BioRender.com. CRISPR: cluster regularly interspaced short palindromic repeats; TALENs: transcription activator-like effector nucleases; ZFNs: zinc-finger nuclease.

The HBV life cycle is illustrated in Figure 2. In brief, the virus enters the host cell by binding to the sodium taurocholate cotransporting polypeptide (NTCP) receptor, which was first identified by Yan et al. [10] in 2012. The viral rcDNA is then imported into the nucleus, where it transforms into the covalently closed circular DNA (cccDNA), intertwining with the host genome. Following transcription, this DNA produces several viral RNAs, such as pgRNA, pre-S/S mRNAs, x mRNA, and pre-core mRNA, which in turn are translated into their corresponding viral proteins. As an intermediate product, pgRNA is reverse-transcribed into progeny viral DNA [11, 12]. The HBx protein is integral for the efficient transcription of cccDNA. Achieving a complete cure for HBV infection is difficult due to the highly stable properties of cccDNA in hepatocytes. Given the complex life cycle of HBV, concerted efforts have been made to explore the antiviral medicines against it. In this paper, we will provide a comprehensive review and discussion of emerging HBV therapies.

## 4. Model System of HBV Infection

### 4.1. Cell Culture Model

Several cell culture systems have been developed for investigating HBV replication and its effects on the liver, as presented in Table 1.

In vitro culture models for HBV research have evolved over decades. Notably, HepG2.2.15 and HepAD38, both derived from HepG2, were the most useful cell models for HBV research [13]. These models cannot be infected with hepatitis B virus naturally. However, they could express HBsAg, HBeAg, and DNA molecules after transfected with cloned HBV DNA in overlength constructs. These cell lines allow for the screening of putative HBV replication inhibitors [16], as well as traditional Chinese medicines (TCM) [14, 15]. MicroRNAs such as miR-185, miR-802, and miR-192-3p, were upregulated in HepG2.2.15, promoting the expression of HBsAg and HBeAg [22, 23] and significantly enhancing HBV DNA replication. Unfortunately, these cell lines fail to embody the attributes of human liver cells. Unlike these cells, HepaRG cells could be differentiated into hepatocyte-like cells (HLCs) and biliary epithelial-like cells. As soon as differentiation, it supports HBV infection and HBV DNA replications. What is more, it contributes to the detection of cccDNA. Yet, the elongated differentiation duration imposes constraints on HepaRG's widespread application [17]. The identification of the NTCP receptor in 2012 enables the understanding of the early life cycle of HBV and contributes to the development of entry inhibitors [10]. Mesenchymal stem cell-derived HLCs exhibit the functions and molecular markers of mature hepatocytes, and their differentiation can be monitored in real-time [18]. Primary human hepatocytes (PHHs) are considered as the most physiologically realistic model for investigating HBV infection [19, 20]. PHHs can be isolated from chimeric mice with humanized livers [21], which support the entire HBV life cycle. The molecular of HBV activity inhibition and related mechanisms were studied in this cell system [24]. Nonetheless, the transient lifespan, poor infection efficiency, and high cost limit the wide application of PHHs. In recent studies, attention has been focused on the generation of hepatic organoids, which promote the dedifferentiation of PHHs into a progenitor state in vitro for subsequent large-scale expansion [25–27]. The development of hepatic organoids will greatly support the study of HBV infection and the corresponding exploration of hepatocarcinogenesis.

### 4.2. Animal Models

Animal models represent an indispensable arsenal for delving into the intricacies of HBV and its host interactions, which are delineated in Table 2.

Chimpanzees are one of the few nonhuman animals with complete immune systems that can support HBV infection. However, ethical concerns restrict studies on chimpanzees. More recently, the rhesus macaque has been developed as a viable HBV animal model, bearing considerable congruence to human hepatic physiology. For the first time in 2017, Burwitz et al. [28] transfected rhesus macaque hepatocytes with Ad- and AAV-based viral vectors that expressed human NTCP; however, the transfection efficiency was low.

The Woodchuck hepatitis virus (WHV) is similar to HBV in terms of viral replication and course of infection. Nearly all chronic WHV-carrier woodchucks develop HCC within 24 months [29]. Young Tupaia (4−9-week-old Tupaia) exposed to HBV develop a chronic infection and exhibit similar hepatic pathological and immunohistochemical changes to humans [30–32]. Furthermore, NTCP was identified as the main HBV receptor in Tupaia hepatocytes [44, 45]

Given the prohibitive costs and ethical constraints associated with the use of the aforementioned animals, mice have become the focal point of research due to their ease of manipulation. Initially, researchers established a transgenic mouse model of HBV genome by pronuclear microinjection of HBV. This model can express various markers of HBV. The HBV transgenic mouse model has markedly contributed to the elucidation of the pathogeny of HBV infection. It is highly useful in assessing antiviral compounds and potential pharmacological agents [33]. However, it cannot detect changes such as liver injury and liver fibrosis in this mouse, and only a few strains of transgenic mice can develop HCC [34, 35]. Subsequently, researchers found that a hydrodynamic HBV DNA injection into the tail vein can also infect the liver, but the infection lasted for only a few weeks [36, 37]. The duration of such infection is extendable by AV/AAV, propelling forward our grasp of the HBV mechanism and the screening of antiviral drugs. Notably, animal age and species are crucial variables affecting transfection efficiency. Additionally, hydrodynamic injections and AdV vectors may provoke innate immune response [38]. The development of the Cre-/Loxp medium in 2014 made the detection of cccDNA a reality [39]. In this regard, cre-transgenic mice injected with a linear HBV genome has been instrumental in the antiviral activities [40, 41] as well as strategies to induce an anti-HBV immune response. In recent years, researchers have constructed humanized chimeric mice by transplanting human hepatocytes or human hematopoietic stem cells into immunodeficient mice [42, 43]. These mice could obtain functional hepatocytes, which has greatly promoted the development of liver regeneration and repair. These chimeric specimens comprising human liver and immune systems are invaluable for probing human-specific insights into HBV infection, immunity, and associated hepatic disease.

## 5. HBV Therapy

The current scope of new drug research for HBV treatment has extended to the entire life cycle of HBV from inhibiting HBV entry to targeting cccDNA and the release of HBV particles, as well as various immune modulation approaches.

### 5.1. Progression in Therapies

For numerous years, the blockade of HBV replication has been the cornerstone of therapeutic strategy development. NAs, such as entecavir (ETV), tenofovir disoproxil fumarate (TDF), tenofovir alafenamide fumarate (TAF), and tenofovir amibufenamide (TMF), are the current mainstays of HBV treatment. NAs are effective in preventing HBV replication because they primarily target viral reverse transcriptase. According to a clinical trial [46], 22% of participants experienced HBeAg loss, and 1% of participants had HBsAg loss after 96 weeks of treatment with TAF at 25 mg among HBeAg-positive patients. Among HBeAg-negative patients, less than 1% of participants have HBsAg loss, a sign of clinical cure of HBV infection. A study [47] from West China Hospital of Sichuan University reveals that TMF has a similar antiviral efficacy to TAF and has no adverse effects on renal function or blood lipids.

Several new NAs are under exploration in the clinical. Pradefovir, a precursor of adefovir, is precisely delivered to hepatocytes. It was applied in a phase II clinical study [48] and inhibited the replication of HBV by inhibiting HBV DNA polymerase. Pradefovir administered in doses of 30, 45, 60, and 75 mg over a span of 24 weeks resulted in HBV DNA reduction of 5.40, 5.34, 5.33, and 5.40 log_10_ IU/mL, respectively, significantly more than the TDF cohort (5.12 log_10_ IU/mL). The highest HBeAg seroconversion, a sign of decreased infectivity of HBV, was obtained with 45 mg of pradefovir with a percentage of 12. In a recent open-label phase II study [49], ledipasvir/sofosbuvir, effective drugs for HCV infection, were applied to HBV infection. Sofosbuvir at 400 mg and ledipasvir at 90 mg for 12 weeks led to an HBsAg decline of 0.4 log_10_ IU/mL in virally suppressed HBV patients and 0.399 log_10_ IU/mL in inactive chronic HBV patients. Sofosbuvir had a HBsAg reduction of 0.207 log_10_ IU/mL, while ledipasvir did not cause HBsAg to drop. Despite the employment of NAs, completely eradicating viral cccDNA remains elusive. HBeAg-positive patients could hardly achieve HBsAg clearance, and only 4%–19% of HBeAg-negative patients could achieve HBsAg clearance with NAs. Most patients with HBsAg positive are prone to relapse upon treatment discontinuation [50].

Despite the above results, the functional cure rate of hepatitis B is still very low with current therapy. The development of effective therapeutic strategies, as well as the discovery of novel antiviral mechanisms, are urgently needed. Compounds targeting HBV entry, cccDNA, nucleocapsid assembly, RNA interference, and others have been studied, as encompassed in the subsequent overview (refer to Table 3).

#### 5.1.1. HBV Entry Inhibitors

Myrcludex B has emerged as a prime drug candidate used for preventing viral entry. It is a synthetic peptide that competes with HBV for the NTCP receptor by imitating the myristoylated pre-S1 region of L-HBsAg [51]. Myrcludex B is demonstrated to block HBV entry efficaciously both in vitro and in vivo [52, 53] which is safe and effective within a period of 24 weeks or at a low dose (2/5 mg) in patients coinfected with HBV and HDV [82].

The potential of vanitaracin A, NTI-007, SCY446, SCY450, and NPD8716 in hindering HBV entry has been evaluated using experimental models [54–57]. Vanitararacin A and NPD8716 are recognized for inhibiting the NTCP transporter function, while NTI-007 is known for tightly binding to NTCP. SCY446 and SCY450, derivatives of cyclosporin A, are found to inhibit HBV entry without inhibiting NTCP transporter activity. Several inflammatory factors, such as IL-1, IL-6, TNF-*α*, and oncostatin M [83–85], are also shown to inhibit HBV entry. In addition, 2H5-A14, a monoclonal antibody, has been shown to suppress HBV entry into hepatocytes by inhibiting the binding of pre-S1 with NTCP [58].

#### 5.1.2. Strategies Targeting Covalently Closed Circular DNA

Currently, the arsenal of antivirals exerting a direct influence on cccDNA remains sparse. Some researchers have constructed recombinant cccDNA (rc-cccDNA) using cre-/loxp-mediated recombinant technology. Rc-cccDNA could persist for a long period following hydrodynamic injection into mice or transfection into hepatocytes. In HepDE19 cells, the production of HBeAg depends on cDNA levels. Therefore, cccDNA levels can be monitored by measuring HBeAg levels. However, the application of this cell line in enzyme-linked immunosorbent assay analyses is limited by the interactions that exist between HBeAg and HBcAg. HepBHAe82, a next-generation cell line, encodes an HA-labeled HBe. It is necessary to develop techniques for monitoring cccDNA with high specificity, sensitivity, and flux, and it is important for the identification of cccDNA-targeting inhibitors.

Ccc_R08, an oral cccDNA inhibitor, is screened through PHHs and HBV circle model mice [59]. When PHHs are treated with ccc_R08 after 2 days of infection, significant reductions were noted in HBV DNA, HBsAg, and HBeAg levels. Additionally, the compound is potent in diminishing replaceable cccDNA in HBV circle model mice.

Ezetimibe, an FDA-approved selective intestinal cholesterol absorption inhibitor, has been shown to prevent the generation of intrahepatic cccDNA and viral replication markers [60].

With the advancements in technology, cccDNA-targeting gene therapy may serve as a potentially curative therapeutic strategy for HBV infection. Gene therapy strategies, such as RNA interference-based gene silencing, epigenetic alteration of target DNA-based genome editing, and cytokine-based immune modulation, based on the aforementioned models, have been developed.

#### 5.1.3. Nucleic Acid Polymers

REP2139, a member of nucleic acid polymers (NAPs), specifically targets the assembly and secretion of subviral particles, thereby hindering the release of HBsAg from infected hepatocytes. When used in conjunction with TDF or TDF + ETV, REP2139 facilitates substantial reductions in both DNA (>3log) and cccDNA (>2log) levels after a 4-week therapeutic period as demonstrated in the duck model [61]. After 48 weeks of follow-up, 14 of 40 patients treated with REP 2139 and TDF and PEGylated interferon alfa-2a acquired HBsAg loss according to a preclinical trial [86].

#### 5.1.4. Capsid Assembly Modulators

HBV capsid assembly modulators (CAMs) are deemed as an attractive approach for new antiviral therapies against HBV. CAMs bind to the capsid site of Cp 183 and affect phosphorylation at eight sites to form aberrant assemblies [87]. Thereby, they can block the assembly of nucleocapsids and disturb the package of pgRNA. CAMs (JNJ56136379) plus NAs for 24 weeks significantly decreased the serum levels of HBV DNA (up to 5.88 log_10_ IU/mL) and HBV RNA (up to 3.15 log_10_ IU/mL) [62]. However, these agents had limited efficacy in HBsAg or HBeAg decline. RO7049389, another agent associated with HBcAg allosteric modulator, is actively undergoing a phase II study [63]. Naïve HBV-infected patients acquire 3.33 log_10_ IU/mL decline in HBV DNA with RO7049389 treatment at 400 mg orally twice a day for 4 weeks [64]. Furthermore, GLS4JHS, NVR 3-778 [65, 66], and AB-423 [67] were also identified to inhibit the core protein of HBV.

#### 5.1.5. RNA Interference or Antisense Oligonucleotide (ASOs)

Small interfering RNA (siRNA), which mainly targets messenger RNAs and pregenomic RNAs, is a promising therapy for hepatitis B infection. According to the clinical trial, small interfering RNA JNJ-3989 plus an NA leads to HBsAg reduction ≥1 log_10_ IU/mL from baseline, which persists in 38% of patients for 336 days after the final treatment [68]. Multiple doses of ARC520 (siRNA) treatment allowed two out of eight patients to acquire the functional cure [69]. A preclinical study reveals that a potent siRNA could decrease the viral RNAs, HBsAg, HBeAg, and HBV DNA levels in a dose-dependent and time-dependent manner when it is encapsulated with RBP131, a novel ionizable lipidoid nanoparticle [88].

Antisense oligonucleotides (ASOs) are single-stranded oligonucleotide, which results in ribonuclease cleavage by forming a complex with HBV RNA. During a phrase IIb trial [70], bepirovirsen (GSK3228836), an antisense oligonucleotide, is injected at 300 mg weekly for 24 weeks among patients receiving NA therapy or not receiving NA therapy. Ultimately, 9–10% of participants with chronic HBV infection acquire the sustained loss of HBsAg (<0.5 IU/mL) and HBV DNA (<20 IU/mL). In a randomized, double-blind study of GSK3389404, HBsAg reduction is up to 1.5log_10_ IU/mL from baseline that occurred in one patient in 60 mg weekly, 120 weekly, and 120 mg biweekly groups [71].

### 5.2. Immunotherapy

**Figure 3:**
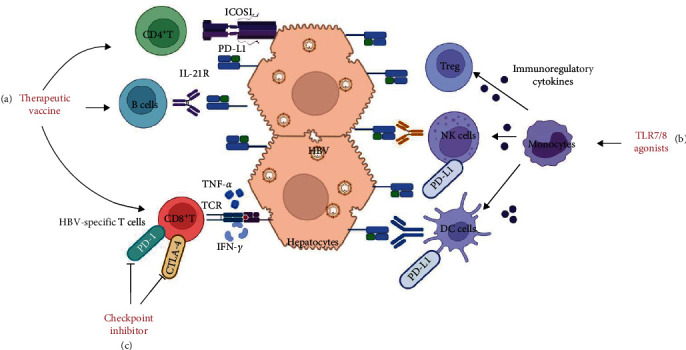
Immunotherapeutic targets. Immunotherapies aim to restore endogenous, depleted HBV-specific CD4^+^and CD8^+^T cells, and memory B cells and restore their function. (a) Therapeutic vaccines could increase the number and function of HBV-specific CD8^+^T, CD4^+^T, and B cells. (b) TLR7/8 agonists can stimulate monocytes to secrete a variety of factors, such as antiviral cytokines to activate HBV-specific CD8^+^T cells, and immunomodulatory cytokines to regulate the function of NK, DC, and Treg cells. (c) Checkpoint inhibitors may partially restore the dysregulated activity of HBV-specific T cells by preventing interactions between PD-1 and PD-L1.

Direct antivirals alone have limited efficacy and are insufficient to restore immune function. Immunotherapy has been applied in clinical practice and had a good immunomodulatory effect on HBV patients (shown in Figure 3).

#### 5.2.1. Interferons

The earliest form of IFN-*α* has been used in the management of HBV infection for more than 30 years. It had also been administered in a modified form to extend the half-life time. PEGylated IFN-*α* (PEG-IFN) is currently the most widely used form. When combined with NA therapy for a duration of 48 weeks, PEG-IFN has contributed to a reduction of qHBsAg by 0.51 log IU/mL in HBeAg-negative patients compared to only 0.14log IU/mL in HBeAg-positive patients [89]. PEG-IFN for over 48 weeks plus ETV and subsequential HBV vaccination in weeks 52, 56, 60, and 76 are applied in CHB patients with undetectable HBV DNA (<20 IU/mL) and HBsAg <3,000 IU/mL. HBsAg loss occurred in 16.2% of patients, which is significantly higher than in patients treated with ETV alone (16.2% vs. 0%, *P*=0.025) [90].

Moreover, regular doses of PEG-IFN supplemented with 24 weeks of low-dose IL-2 were able to increase the number and function of HBV-specific CD8^+^T cells, which in turn promotes the conversion of HBeAg [91]. PEG-IFN could not only directly suppress HBV replication but also boost the NK cell response [92, 93]. Based on the findings of previous studies, antiviral cytokines can also eliminate cccDNA [94]. Nevertheless, the side effects associated with PEG-IFN should be of concern.

#### 5.2.2. Therapeutic Vaccines

The introduction of the hepatitis B vaccine has greatly reduced the incidence of HBV infection over the past few decades. Alongside this, the progression of therapeutic vaccines has been integral to the HBV therapeutic landscape.

Engerix-B, as a therapeutic hepatitis B vaccine, has progressed a phase II clinical trial. In patients with occult HBV infection, sequential Engerix-B inoculation at weeks 0, 12, 24, and 36 has significantly increased the number of CD8^+^T and B lymphocytes in the peripheral blood [72]. GS-4774 vaccine was designed to induce HBV-specific T-cell responses. It is usually injected every 4 weeks up to week 20, including 6 doses in total, with 2, 10, or 40 yeast units. A phase II trial demonstrated that 5 out of 151 CHB patients receiving GS-4774 achieve HBeAg seroconversion. Three out of 50 participants receiving 40 yeast units dosage of GS-4774 experienced HBsAg decline ≥0.5log_10_ IU/mL [73]. Moreover, GS-4774 has been shown to boost the secretion of IFNG, TNF, and IL2 from CD8^+^T cells in patients not undergoing any concurrent treatment [74]. Additionally, a novel nanoparticle lipopeptide vaccine and six doses of subcutaneous injection of 900 *μ*g _*Ɛ*_PA-44 resulted in HBeAg serological conversion in 38.8% of HBeAg-positive patients [75]. Contrasting these advancements, another clinical trial reveals that five doses of anti-HBV DNA vaccination cannot inhibit reactivation after discontinuation of NAs in hepatitis B patients [95]. HBV-specific CD8^+^T and CD4^+^T cells are significantly enhanced with the vaccine [76].

#### 5.2.3. Agonist of Receptor

The pursuit of receptor agonists to modulate immune response in HBV treatment has led to notable discoveries.

RO7020531, an agonist for toll-like receptor7 (TLR7), has been trialed across a dosage spectrum of 3–170 mg. The safety profile of RO7020531 is favorable, indicating that it is well-tolerated in HBV patients [77]. The activation of toll-like receptor 8 (TLR8) contributes to the functional cure of HBV infection. Selgantolimod, a TLR8 agonist, has been studied in a phase Ib trial [78]. This study administers selgantolimod in doses of 1.5 or 3 mg for virally suppressed patients and 3 mg for viremic patients, taken orally once a week over a 2-week span. It is safe and well tolerated in patients with CHB. Notably, selgantolimod has the capability to activate natural killer (NK) cells, dendritic cells (DC), and HBV-specific CD8^+^ Tcells by eliciting many cytokines when it is administered to human peripheral blood monocyte cells of patients with CHB in vitro [79]. Furthermore, inarigivir, a retinoic acid-inducible gene I agonist, is administered ranging from 25 to 200 mg for 12 weeks. The levels of HBV DNA and RNA are significantly decreased after treatment in naïve patients [80].

#### 5.2.4. Checkpoint Inhibitor

The exploration of checkpoint inhibition has been scrutinized through detailed in vitro and in vivo study designs. These checkpoint inhibitors were found to partially restore the dysregulated activity of HBV-specific T cells by preventing interactions between PD-1 and PD-L1. Nivolumab, a PD-1 inhibitor, was effective in the treatment of advanced hepatocellular carcinoma [96, 97]. It can reverse T-cell depletion, enhance the body's cellular immunity, and promote the attack of activated T cells against tumor cells. Anecdotal evidence suggests that nivolumab might restore HBV-specific T cells in HBV-infected patients. A noteworthy proportion of patients experiences a decrease in HBsAg levels, with one patient achieving HBsAg loss when nivolumab is administered at baseline and week 4 [81].

### 5.3. Traditional Chinese Medicine

In China, TCM is widely used in the treatment of HBV infection, mainly for immune modulation and symptom reduction, which is summarized in Table 4. TiaoGanJianPiJieDu granule combined with ETV leads to the negative conversion of HBeAg in 37.54% of patients at week 108 [98], significantly higher than that in the control group (*P*=0.008). HBV DNA levels declined from 6.54 ± 0.95 (log) to 5.15 ± 2.22 (log) in the Bushen formula combined ETV group after 6 months of treatment [99], significantly more than the single ETV group (*P* < 0.05). Further analysis suggests that the result was correlated with increasing the frequency of Th1 and DC cells and lowering the frequency of Treg cells [100]. The latest study revealed that Bushen formula decreases the HBeAg levels and activates the differentiation of T cells and B cells in CHB patients [101]. Additionally, TCM plays an obvious advantage in blocking the progression of fibrosis/cirrhosis. Some TCM formulas, such as Anluohuaxian pills, Fuzheng Huayu tablet, and Biejia Ruangan compound, can prevent the progression of CHB to liver fibrosis and liver cancer [104, 109]. In patients with hepatitis B cirrhosis, the combination of Fuzheng Huayu tablet and ETV did not alter the antiviral efficacy of ETV; however, it enhanced the rate of biochemical response and had a tendency to improve the rate of serological conversion of HBeAg and liver fibrosis [102, 103]. Based on a recent study, Biejia Ruangan compound with combined antiviral therapy can reduce the incidence of HCC in CHB patients [105].

In addition to herbal medicine, TCM techniques such as acupuncture, acupoint injection, and moxibustion also contribute to the treatment of HBV infection. Acupuncture at Waiguan (GV 6), Zusanli (ST 36), Yanglingquan (GB 34), and Sanyinjiao (SP 6) combined with ETV can contribute to the reduction of HBV DNA and HBsAg in CHB patients. Astragalus injection and IL-2 were applied to CHB patients through acupuncture point injection. Astragalus injection at Zusanli (ST 36) combined with NA treatment for 12 months increases the rate of HBeAg-negative conversion compared with patients in the single NA group (*P*  < 0.05) [106]. Acupuncture point injection of interferon in CHB patients compared to subcutaneous injection significantly increases the negative conversion rates of HBsAg, HBeAg, and HBV DNA [107]. Research suggests that treatment of moxibustion in CHB patients reduces patients' anxiety scores and improves their sleep quality scores [108]. Acupuncture adjuvant treatment also improves liver function and increases the percentage of CD4^+^ T cells in patients with hepatitis B cirrhosis [110].

## 6. Conclusion

HBV cell culture systems and HBV animal models have offered significant opportunities for research on the mechanism of HBV infection, which contributes to exploring more effective antiviral therapeutic strategies. In addition, the development of liver organoids will provide more useful methods to screen antiviral drugs. Furthermore, TCM has been confirmed to be an effective treatment for CHB patients by regulating the immune status of the patient, which will provide the potential for the development of new drugs.

While antiviral drugs can reduce HBV DNA levels, current treatments typically fail to eradicate viral cccDNA. The main obstacles to antiviral strategies include the presence of cccDNA, the substantial changes in HBV-specific immunity, and the presence of embedded DNA. Blocking the entry of HBV into hepatocytes, clearing cccDNA, and fine-tuning immune regulation may be potential strategies in the future.

## Figures and Tables

**Table 1:** Cell models of HBV infection.

System	Cell source	HBV genome background	Advantage	Limitation	Application	Reference
HepG2.2.15	HepG2/transfected	pDolTHBV-1 plasmid vector containing two head-to-tail copies of HBV genome	Stable express HBV particles	Limited HBV production	Evaluate the effects of conventional antiviral medications Investigate HBV-associated HCC Investigate inflammatory factors	[13, 14]

HepAD38	HepG2/transfected	0.3 *μ*g pUHD15-1neo and 2.7 *μ*g ptetHBV (single pgRNA)	Support formation of cccDNA High-level production	—	Screen HBV replication inhibitors Support immune-related studies	[15]

HepG2-NTCP	HepG2/infection	—	Support infection High level of HBV	Only partially mimic normal hepatocytes No spread of HBV	Support screening of antiviral medications Molecule-associated HBV entry	[16]

HepRG	HepRG/infection	—	Mimic normal hepatocytes DMSO restriction	Long differentiated time	—	[17]

Hepatocyte-like cells	Human pluripotent stem cells Human embryonic stem cells/infection	—	Quantity production Support entire life cycle Naturally express NTCP Spread of HBV	Low replication efficiency	Support high-throughput screening of antiviral medications Screen personalized antiviral medications	[18]

Primary human hepatocytes	Primary human hepatocytes/infection	—	Mimic normal hepatocytes the best	Expensive, scarce, limited life span	Screen antiviral molecule and related mechanisms Investigate interactions between virus and host hepatocytes	[19–21]

*cccDNA*, covalently closed circular DNA.

**Table 2:** Animal models of HBV infection.

Animal	Subtype	cccDNA formation	Feature/application	Limitation	Immune response	Reference
Nonhuman primate experimental model	—	Yes	Age and dose dependent	Ethical limitation	Immune-competent T-cell immune response	[28]

Woodchuck	—	Yes	Develop HCC Age and dose dependent	—	Immune-competent B- and T-cell immune response	[29]

Tupaia	—	Yes	Support the discover of NTCP Age dependent	—	Immune-competent	[30–32]

Transgenic mice	—	Yes	Expressing HBV particles Integrated HBV genome Assess antiviral compound	Natural immune tolerance Not develop liver disease	Innate and adaptive immune response	[33–35]

Transfected mice	Hydrodynamic injection	No	HBV only last for several weeks	No liver damages	Innate immune response	[36–41]
	AdV/AAV injection	No	Stable infection Strain and age dependent	No liver damages	Innate and adaptive immune response	
	Recombinant cccDNA	Yes	—	Few develop fibrosis Short duration	Innate and adaptive immune response	

Humanized chimera mice	uPA/SCID mice	Yes	Higher grafting survival rate	Immunodeficient	—	[42, 43]
	FRG chimera	Yes	Could transplanted with hematopoietic cells	Immunodeficient	+/−	
	AFC-hu HSC/Hep mice	Yes	Human immune system Develop liver diseases	—	Innate and adaptive immune response	

*AdV*, adenovirus; *AAV*, adeno-associated virus-based vectors; *cccDNA*, covalently closed circular DNA; *HCC*, hepatocellular carcinoma; *HSC*, hematopoietic; *NTCP*, sodium taurocholate cotransporting polypeptide.

**Table 3:** Novel HBV therapeutics in development.

Drug name	Mechanism/efficacy	Phase	Reference
Nucleot(s)ide analogs			
Entecavir	Inhibit HBV DNA polymerase	On-market	—
Tenofovir disoproxil	Inhibit HBV DNA polymerase	On-market	—
Tenofovir alafenamide	Inhibit HBV DNA polymerase	On-market	[46]
Tenofovir amibufenamide	Inhibit HBV DNA polymerase	On-market	[47]
Pradefovire	Inhibit HBV DNA polymerase	Phase III	[48]
Sofosbuvir	Nucleotide polymerase inhibitor	Phase II	[49]
Entry inhibitors			
Myrcludex B	Competitive binding of NTCP with HBV	Phase II	[51–53]
Vanitararacin A	Inhibit NTCP transporter activity	Preclinical	[54]
NPD8716	Inhibit NTCP transporter activity	Preclinical	[55]
NTI-007	Bind with NTCP	Preclinical	[56]
SCY446	Interact directly with NTCP to inhibit viral attachment to host cells	Preclinical	[57]
SCY450	Interact directly with NTCP to inhibit viral attachment to host cells	Preclinical	[57]
2H5-A14	Inhibit the binding of pre-S1 with NTCP	Preclinical	[58]
cccDNA-targeting strategies			
Ccc_R08	Decrease the level of HBV DNA, HBsAg, HBeAg, and cccDNA	Preclinical	[59]
Ezetimibe	Cholesterol absorption inhibitor, prevent generation of cccDNA	Preclinical	[60]
Nucleic acid polymers (NAPs)			
REP2139	Target the assembly and secretion of subviral particles and inhibit the release of HBsAg, increase rate of HBsAg loss	Phase II	[61]
Capsod assembly modulators (CAMs)			
JNJ56136379	Block the assembly of nucleocapsid and disturb the package of pgRNA	Phase II	[62]
ABI-H0731	Core protein inhibitor	Phase II	—
RO7049389	Core protein allosteric modulator	Phase II	[63]
AB-506	Core protein biding	Phase I	[64]
ABI-H2158	Core protein biding	Phase I	—
GLS4JHS	Core protein biding	Phase II	[65]
NVR 3-778	Induce the formation of capsid-like particles	Phase I	[66]
AB-423	Inhibit encapsidation of pgRNA	Preclinical	[67]
RNA interference			
JNJ-3989	RNA degradation	Phase IIa	[68]
ARC520	RNA degradation	Phase Ib	[69]
GSK3228836 (ASOs)	Result in ribonuclease cleavage	Phase II	[70]
GSK3380404 (ASOs)	Result in ribonuclease cleavage	Phase II	[71]
Immunotherapy			
*Therapeutic vaccines*			
Engerix-B	Increase the number of CD8^+^T and B lymphocytes	Phase II	[72]
GS-4774	Induce HBV-specific T-cell response	Phase II	[73, 74]
_*Ɛ*_PA-44	Nanoparticle therapeutic vaccine, 38.8% of patients had HBeAg serological conversion	Phase III	[75]
ChAdOx1 HBV	T-cell vaccine	Phase I	[76]
* Agonist of receptor*			
RO7020531	TLR7 agonist	Phase I	[77]
RO7011785	TLR7 agonist	Phase I	—
Selgantolimod	TLR8 agonist, activate NK, DC, and HBV-specific CD8^+^T cells	Phase Ib	[78, 79]
Inarigivir	Retinoic acid-incucible gene I agonist, decrease HBV DNA and RNA	Phase II	[80]
* Checkpoint inhibitors*			
Nivolumab	Anti-PD1	Phase Ib	[81]
Cemiplimab, REGN2810	Anti-PD1	Phase I/II	—

ASOs, antisense oligonucleotide; cccDNA, covalently closed circle DNA; DC cells, dendritic cells; NKs, natural killercells; NTCP, sodium taurocholate cotransporting polypeptide; PD1, programmed death 1.

**Table 4:** Therapies of traditional Chinese medicine.

Therapies	Efficacy	Phase	Reference
TiaoGanJianPiJieDu granule	Increase the negative conversion of HBeAg	Clinical experience	[98]

Bushen formula	Increase the frequency of Th1and DC cells, decrease the HBV DNA level and the frequency of Treg cells Decrease the HBeAg level and activate the differentiation of T cell and B cell	Clinical experience	[99–101]

Fuzhenghuayu tablet	Improve the rate of serological conversion of HBeAg and liver fibrosis	On-market in China	[102, 103]

Anluohuaxian pills	Prevent progression to liver fibrosis and liver cancer	On-market in China	[102]

Biejia Ruangan compound	Reduce the incidence of HCC	On-market in China	[104, 105]

Acupuncture	Increase the rates of HBeAg-negative conversion Increase the percentage of CD4^+^ cells	Clinical experience	[106]

Acupuncture point injection	Increase the rates of HBeAg conversion and HBV DNA conversion	Clinical experience	[107]

Moxibustion	Reduce patients' anxiety score and improve their sleep quality score	Clinical experience	[108]

## Data Availability

Data sharing does not apply to this article as no new data were created or analyzed in this study.
